# Chronic stressors and burnout in Dutch police officers: Two studies into the complex role of coping self-efficacy

**DOI:** 10.3389/fpsyg.2022.1054053

**Published:** 2022-12-16

**Authors:** Liselotte Marina Josephine Eikenhout, Roos Delahaij, Karen Van Dam, Wim Kamphuis, Inge Leonie Hulshof, Joris Van Ruysseveldt

**Affiliations:** ^1^Faculty of Psychology, Open University of the Netherlands, Heerlen, Netherlands; ^2^Human Behaviour & Training, Netherlands Organisation for Applied Scientific Research (TNO), Amsterdam, Netherlands

**Keywords:** burnout, coping self-efficacy, police officers, conservation of resources, resource depletion

## Abstract

**Introduction:**

Burnout complaints are high for Dutch police officers. According to Hobfoll’s Conservation of Resources theory, resources such as coping self-efficacy can play an important role in the burnout process. The aim of this study was to investigate the buffering effect of coping self-efficacy on burnout, as well as a possible depletion effect of burnout on coping self-efficacy.

**Methods:**

As such, this research consists of two studies namely, a two-wave study (*N* = 166) and three-wave study (*N* = 95) on Dutch police officers. They expand on previous research regarding coping resources and police burnout.

**Results:**

Both studies show that the chronic stressor, work scheduling, was positively associated with burnout. Also, coping self-efficacy weakened the effect of work scheduling (Study 1) and workload (Study 2) on burnout. Moreover, there was a direct negative relationship between burnout and coping self-efficacy.

**Discussion:**

The results indicate that burnout can lead to lower coping resources, initiating a potential cycle of resource loss and burnout. Further investigation into this depletion effect is required to provide police officers and organisations with tools to prevent burnout.

## Introduction

Police officers (POs) continuously encounter stressful situations, which may put their own lives at risk. Police work is considered a high-risk profession consisting of ongoing chronic work demands, which have been found to impact POs wellbeing. This can have cascading effects for individuals, the police organisation, and society at large. For example, chronic stressors negatively impact health outcomes such as burnout (Folkman et al., 1986; Bakker and Heuven, 2006; Burns et al., 2016; Queirós et al., 2020) and cardiovascular disease (Lepore et al., 1997; Goldstein and Kopin, 2007). Furthermore, ongoing demands can negatively influence motivation, engagement, and job performance and subsequently lead to increased (long-term) absenteeism and turnover intentions amongst POs (Anshel, 2000; Kop and Euwema, 2001; Schaufeli et al., 2009; Demerouti and Bakker, 2011). Additionally, POs can show increased aggressive reactivity (e.g., brutality) or indifference towards civilians (Abdollahi, 2002; Queirós et al., 2013; NOS, 2019), leading to poor police-community relations.

According to Conservation of Resources (COR) theory, resources reduce the impact of stressors (Hobfoll, 1989; Hobfoll et al., 2018). Research has shown that resources have a buffering effect on the relationship between demands and wellbeing (e.g., adopting the Job Demands-Resources (JD-R) model; Schaufeli et al., 2009; Demerouti and Bakker, 2011). However, prolonged or repeated exposure to stressors can have negative effects on individuals’ coping resources and initiate a resource depletion process (Hobfoll, 1989; Westman et al., 2004). For example, burnout can affect an individual’s self-efficacy to cope with stressors (Llorens-Gumbau and Salanova-Soria, 2014), which in turn impedes effective coping in the future resulting in more burnout-related complaints. A possible explanation is that self-efficacy buffers the effects of demands on burnout. Although the relationship between chronic stressors and wellbeing have been extensively studied (e.g., Eckenrode, 1984; Lee and Ashforth, 1996; Igic et al., 2017; Keller et al., 2020), research on the long-term negative effects of stressors on coping resources and depletion processes is scarce, especially in police contexts.

The aim of this study is to investigate the complex role of coping self-efficacy in a context where chronic stress is prevalent (i.e., the police force) using COR theory as our theoretical framework. This study contributes to the literature in three ways. First, our goal is to study the process of resource depletion in addition to resources’ potential buffering effect. Relationships between chronic stressors and wellbeing have been extensively researched (e.g., Eckenrode, 1984; Lee and Ashforth, 1996; Igic et al., 2017; Keller et al., 2020), as well as the buffering role of self-efficacy (Bandura, 2001; Llorens et al., 2007; Lan et al., 2020; Zeng et al., 2020). Also, resources and self-efficacy have been studied in terms of gain cycles (Llorens et al., 2007), thereby confirming COR theory’s tenet that individuals strive to gain resources (Hobfoll, 1989). However, research on the long-term negative effects of stress on coping resources and a potential depletion process is scarce. Since coping self-efficacy plays a key role in preventing or reducing stress, it is crucial to investigate a possible depletion of this resource.

Second, although many studies investigated generalised self-efficacy, this study focuses on coping self-efficacy (CSE) as a relevant personal resource. CSE relates to individuals’ beliefs about their ability to cope with stressful situations (Delahaij and Van Dam, 2017) and includes beliefs on stress resilience and stress recovery. Although theoretical models such as Bandura’s (1997) social cognitive theory and Lazarus’ (1991) Appraisal theory emphasise the importance of individuals’ CSE for their affective responses and wellbeing, this CSE construct has been underresearched within the work context. Given our dynamic 24/7 economy, technological developments and high work demands, CSE is an important personal resource for all employees, and for high-risk professionals, such as the police. Therefore, gaining more insights into the dynamics of CSE and burnout symptoms is not only of theoretical interest, but also holds practical value. Especially since the police in Netherlands consists of over 50,500 operational employees (at the end of 2020; Politie, 2021).

Finally, to test the research model, our study has a longitudinal approach using both a two-wave and a three-wave design. Research in the police context has predominantly used a cross-sectional design (Violanti et al., 2017), which excludes causal interpretations of the studied associations. A longitudinal approach is more suitable when studying the temporal processes of (chronic) stress and burnout, and the long-term adverse effects on coping resources. Longitudinal study designs can help determine causality, which is fundamental in depletion processes. This study aims to fill this gap in the stress literature, by incorporating multiple relevant stressors, CSE, and burnout for POs in two study designs. To validate the research model, it was tested on two independent samples.

### Conservation of resources

Coping resources are any physical, psychological, social, or organisational factors that help an individual cope with stressors (derived from job resources definition; Demerouti and Bakker, 2011) and play a key role in the stressor-strain relationship. Hobfoll’s (1989) COR theory states that individuals try to retain, protect, and build resources and a (potential) loss of these resources threatens them. Although successful resource gain can lead to positive wellbeing, a lack of resource gain or (potentially) losing resources due to (environmental) stressors can cause psychological stress (Hobfoll, 1989; Westman et al., 2004). Ongoing psychological stress can result in burnout and add to the wear and tear of coping resources, initiating a resource depletion process (Westman et al., 2004; Hobfoll et al., 2018). In short, using resources will reduce the impact of demands and in turn prevent or reduce stress. However, ineffective coping and ongoing distress can deplete these resources causing a loss cycle during stressful circumstances (Hobfoll, 1989). Hence, exposure to continuing (i.e., chronic) stressors might be especially detrimental to the resources over time. Ultimately, we wish to investigate whether a loss cycle of resources exists, and if so, whether we can prevent the onset of such a cycle. Therefore, we will examine the effects of chronic stressors on burnout (H1), the buffering effect of resources on this relationship (H2), and the depleting effect of burnout on resources (H3).

### Effects of chronic stressors on burnout

Continuous psychological distress can lead to burnout and can wear down coping resources. Burnout is defined as a slow-developing, long-term, work-related condition and is primarily characterised by exhaustion, dysregulation of emotional and cognitive processes, and mental distancing (i.e., depersonalization or cynicism; Schaufeli et al., 2019). Burnout is a result of an imbalance between high demands and insufficient resources (Schaufeli and Taris, 2014), that is, when chronic stressors exceed the ability to cope with these stressors effectively.

Chronic stressors are long-term, ongoing stressors that people encounter daily, and have a high chance of recurrence (Eckenrode, 1984; Lepore et al., 1997; Day and Livingstone, 2001). Examples of chronic stressors that generally occur in POs’ work environment (Kamphuis et al., 2014) and have been linked to burnout symptoms include: high workload, work scheduling (including irregular work and task division), and interruptions (e.g., Demerouti et al., 2004; Peterson et al., 2019; Puranik et al., 2020). Workload refers to the work and task demands placed on an individual (Van den Broeck et al., 2010) and is generally associated with a risk of impaired wellbeing and decrements in performance and willingness to perform (Meijman and Mulder, 1998). Previous studies indicate that Dutch POs face high workload, which has resulted in high sickness absence rates (Huijs et al., 2014; Hooftman et al., 2018). Many studies have shown a strong relationship between workload and burnout symptoms (Lee and Ashforth, 1996;Demerouti et al., 2004; Van den Broeck et al., 2010). For instance, workload is positively associated with emotional exhaustion (Demerouti et al., 2004; Van den Broeck et al., 2010) and cynicism (Taris et al., 2017).

The police are on standby 24/7, which makes shift work and irregular working hours unavoidable. Work scheduling refers to circumstances, such as top-down scheduling decisions concerning the planning of working hours, including shift work, irregular working hours, and unfair task divisions. Shift work, irregular working hours, and sudden changes in POs’ schedules are inherent to police work and are associated with increased stress (Ma et al., 2015), work dissatisfaction and sleep complaints (Gerber et al., 2010), reduced stress resilience (Violanti et al., 2017), and burnout (Peterson et al., 2019).

Additionally, the work of POs is characterised by interruptions and shifting focus, largely due to variation and timing of police reports and PO duties. A work interruption can be defined as a suspension of behavioural performance, attentional focus, or both from an ongoing work task (Puranik et al., 2020). Interruptions can hinder attentional processes, impede goal progress, trigger affective reactions, and deplete resources (Lin et al., 2013; Beck et al., 2017). This can result in cognitive dysregulation as attentional resources are diverted from the ongoing task (Puranik et al., 2020). Also, impeded goal progress due to disturbances is associated with frustration, low levels of enthusiasm and commitment (Beck et al., 2017), thereby increasing the risk of burnout symptoms.

The aforementioned chronic stressors are common for police work: like acute stressors, such as physical threats and emotional demands, chronic stressors are part of police work. These demands constantly tap into coping resources and could therefore initiate resource loss over time. Therefore, the following hypothesis is proposed:

*Hypothesis 1*: Workload (H1a), work scheduling (H1b), and interruptions (H1c) positively relate to burnout.

### The dual role of coping self-efficacy

Coping self-efficacy is an important resource for coping with stress. CSE is an individual resource defined as an individual’s belief in their ability to effectively cope with specific stressful situations, which strongly influences their appraisal of a stressor (Delahaij and Van Dam, 2017). People with high levels of CSE perceive situations as less threatening and more challenging, because they consider the situation to be more controllable (Folkman and Lazarus, 1985; Delahaij et al., 2011). Moreover, studies have shown that high CSE individuals adopt more effective strategies to cope with stress (Bandura, 2001; Benight and Harper, 2002; Delahaij et al., 2011; Delahaij and Van Dam, 2017), which in turn leads to less strain and exhaustion. For example, Delahaij et al. (2011) found that individuals with high CSE are more likely to show task-focused behaviour (vs. emotion-focused behaviour), turning a dangerous situation into a more benign situation. This could reduce the strain placed on individuals. On the other hand, individuals with a low sense of self-efficacy are more likely to experience taxing work situations as stressful (Bandura, 1997). Indeed, CSE is an important predictor of distress and job satisfaction (Anshel, 2000; Benight and Bandura, 2004; Stetz et al., 2006; Smith et al., 2013). Thus, CSE has been established as a resource that buffers the stressor-strain relationship. This is also in line with research into the JD-R model, which states that resources act as a buffer in the health impairment process (Schaufeli et al., 2009; Demerouti and Bakker, 2011). To investigate the buffering effect of CSE for the previously specified chronic stressors, the following hypothesis is proposed:

*Hypothesis 2*: Coping self-efficacy moderates the positive relationships between chronic stressors and burnout. Specifically, the relationship between chronic stressors and burnout is weaker when coping self-efficacy is high (vs. low).

The tenets of COR theory propose mechanisms besides the traditional hypothesis of the buffering effects of resources on outcomes. COR theory helps explain both gain and loss cycles of resources (Westman et al., 2004). Those who have sufficient resources are able to build or gain resources more easily by investing their resources. Conversely, individuals who have a lack of resources are vulnerable to additional loss. This study focuses on the depletion element of the latter. We propose that CSE plays a key role in coping with stress through a potential depletion process (Bandura, 2001; Llorens et al., 2007). CSE is an efficacy belief that can degrade as a result of ongoing stress in combination with ineffective coping. According to Bandura (1997), efficacy beliefs can be enhanced through mastery experiences. However, a lack of mastery can also degrade these beliefs. This will lead to further negative appraisal of their ability to cope with stress and in turn increase the impact of organisational stressors on an individual’s physical and emotional health.

Thus, the constant adjustments to chronic stressors can lead to wear and tear of coping resources, which may result in a loss spiral (Hobfoll et al., 2018). Efficacy beliefs (i.e., personal resource) develop from several sources, such as affective states and earlier coping experiences (Bandura, 2001; Benight and Bandura, 2004). For example, negative experiences in dealing with work scheduling (i.e., ineffective coping) can cause negative emotions to predominate, which leads to dissatisfaction, sleep complaints (Gerber et al., 2010), and burnout (Peterson et al., 2019). In turn, individuals can feel less confident in coping with similar situations in the future (i.e., reduced efficacy beliefs) due to the negative associations with this stressor. As a result of these reduced personal resources, individuals may show less proactive behaviour in preventing burnout (Otto et al., 2021). Work obstacles can lead to exhaustion of resources over time due to overtaxing (Llorens-Gumbau and Salanova-Soria, 2014). Overtaxing depletes energy and leads to burnout and negative affective states, which in turn causes a reduction in self-efficacy (Llorens-Gumbau and Salanova-Soria, 2014). To summarise, CSE has a moderating effect on the stressor-strain relationship and can be depleted as an outcome of the coping process. Since CSE is crucial for managing stress and facilitating coping processes, it is critical to investigate this dual role. To investigate the second role of CSE (i.e., as an outcome of the stress process), the following hypothesis is proposed:

*Hypothesis 3*: Burnout has a negative relationship with coping self-efficacy.

### Aim of the studies

The aim of this study is to investigate the complex role of CSE in the stress process, by focusing on both a buffering effect and a resource depletion process. The proposed hypotheses (i.e., H1, H2, and H3) were tested in two independent samples, using a two-wave design (Study 1) and a three-wave design (Study 2). Multiple-wave designs were used to investigate the long-term effect of chronic stressors, CSE, and burnout symptoms on future CSE. Study 2, taking into account an even longer period of time (i.e., three consecutive years), was performed to validate the results of Study 1 and our research model.

## Study 1

### Materials and methods

#### Participants and procedure

Study 1 was designed to use a two-wave online questionnaire, which was administered at a police district in a large city in Netherlands. Two samples were collected at separate time intervals and were combined into one dataset. For the first sample, Time 1 was in May 2019 and Time 2 was in March 2020. For the second sample, Time 1 was in March 2020 and Time 2 was in March 2021. This study was approved by TNO’s internal ethical committee (registration number: 2018-075): Research participants were treated in accordance with the ethical guidelines set out by the American Psychological Association (2017). Pseudonyms were used to track participants over time. Participants received information on the questionnaire’s process and goals, anonymity, and confidentiality. Subsequently, participants gave their informed consent by actively ticking a box to begin the questionnaire.

In May 2019, POs were approached through flyers and human resources. Participants registered through a designated email address. In March 2020 and March 2021, the POs were approached directly through email. Several reminders were sent to increase the response rate. Only participants who completed two consecutive waves were included in this study, resulting in a sample of 166 participants (*N*_sample 1_ = 43, *N*_sample 2_ = 123). Majority of the POs were over 30 years old (45% between 30 and 45; 40% older than 45 years). Most had received vocational education (49%; 18% higher education; 31% high school). Gender was not registered to reduce traceability to individuals within their teams.

#### Measures

Unless indicated otherwise, a five-point scale from 1 (never) to 5 (always) was used. Workload was measured with three items of the Police Resilience Monitor (PRM), a measure for POs that was developed based on literature study, interviews, focus groups, and validated and published scales (Kamphuis et al., 2014).^1^ An example item for workload is “It happens that I am unable to finish my work.” Cronbach’s alpha was 0.74 (T1) and 0.74 (T2).

Work scheduling was measured with five items of the PRM (Kamphuis et al., 2014). The items are related to scheduling, irregular working hours, and fairness of task division. An example item is “Indicate to what extent you have experienced stress during the past 12 months due to the way scheduling takes place.” Cronbach’s alpha was 0.75 (T1) and 0.70 (T2).

Interruptions was measured with two items from the PRM (Kamphuis et al., 2014). An example item is “Indicate to what extent you have experienced stress during the past months due to being interrupted by others during my work.” Cronbach’s alpha was 0.79 (T1) and 0.73 (T2).

Burnout was measured with 10 items: Five items from the National Working Conditions Survey (NEA), which is based on the Utrecht Burnout Scale (Hooftman et al., 2018) for emotional exhaustion, and five items from the Utrecht Burnout Scale for Contact Professions (UBOS-C; Schaufeli and Van Dierendonck, 1995) for depersonalization. Answers were rated on a seven-point scale, ranging from 1 (never) to 7 (every day). Example statements are “I feel completely exhausted because of my work” and “I do not really care about what happens to some people.” Cronbach’s alpha was 0.87 (T1) and 0.82 (T2).

According to Bandura (1997), generalised efficacy measures are weak predictors of person-and situation-specific behaviour and outcomes, in contrast to tailored efficacy measures. Since the focus of this study was to identify outcomes of chronic stressors, we measured coping self-efficacy. CSE was measured with 10 items from the PRM (Kamphuis et al., 2014). The items are related to stress resiliency, stress recovery, and trust in oneself. An example item is “I trust my own skills as a police officer.” Cronbach’s alpha was 0.87 (T1) and 0.89 (T2).

Demographic variables (age and education) were measured categorically to limit the possibility of traceability to individuals within the district. For age, the answer options were younger than 30, between and including 30 and 45, older than 45, and do not wish to disclose. For education, answer options were based on the Dutch education system; high school (i.e., VMBO, HAVO, and VWO); vocational education (i.e., MBO); higher education (i.e., HBO and WO); and do not wish to disclose.

#### Statistical analysis

Confirmatory Factor Analysis (CFA) was conducted to examine measurement models of our research variables. The fit indices of the model, in which each variable in the research model was represented by a separate latent factor (i.e., workload, work scheduling, interruptions, CSE, and burnout), indicated a good fit (*χ*^2^(384) = 539.64, CFI = 0.93, TLI = 0.92, RMSEA = 0.05). The hypotheses were tested with a bootstrapping analysis (Preacher et al., 2007) using Hayes’ Process 3.4.1 macro (model 7, moderated mediation) with 5.000 resamples (Hayes, 2018). Model 7 is a moderated mediation model in which the moderator only moderates the relationship between the independent variable and the mediator. Process’ model 7 allows all hypotheses to be tested in one model. CSE T2 was the dependent variable, stressors T1 were the main predictors, CSE T1 was the moderator, and burnout T1 was the mediating variable. Separate analyses were conducted for each predictor and the other stressors were included as covariates to ensure all interaction variables were analysed. The analyses were repeated with burnout T2 as the mediating variable to confirm the long-term effects of stressors and CSE on burnout. The analyses were performed in SPSS 25.

### Results

The means, standard deviations, Cronbach’s alpha coefficients, and bivariate correlations (including test–retest correlations) of the scales at T1 and T2 are presented in Table 1. Moderate to strong internal consistencies were found for the scales in this study (0.70–0.89). Correlations between the chronic stressors workload, work scheduling, and interruptions are moderate to large (Haslam and McGarty, 2003). As expected, burnout was significantly correlated to the stressors (positive) and CSE (negative) at both timepoints.

**Table 1:** Descriptive statistics Study 1 (scale, mean, standard deviation, Cronbach’s alpha, and Pearson correlations).

	Range	Mean	SD	*n* items	α	1	2	3	4	5	6	7	8	9
1. T1 Workload	1–5	2.59	0.72	3	0.74									
2. T1 Work scheduling	1–5	2.47	0.82	5	0.75	0.44***								
3. T1 Interruptions	1–5	2.60	1.04	2	0.79	0.57***	0.53***							
4. T1 Coping self-efficacy	1–5	3.96	0.44	10	0.87	−0.18*	−0.28***	−0.23**						
5. T1 Burnout	1–7	2.00	0.84	10	0.87	0.31***	0.49***	0.40***	−0.42***					
6. T2 Workload	1–5	2.51	0.71	3	0.74	0.63***	0.32***	0.41***	−0.11	0.21**				
7. T2 Work scheduling	1–5	2.47	0.73	5	0.70	0.35***	0.65***	0.39***	−0.22**	0.42***	0.35***			
8. T2 Interruptions	1–5	2.67	0.96	2	0.73	0.39***	0.37***	0.55***	−0.26***	0.26***	0.46***	0.53***		
9. T2 Coping self-efficacy	1–5	4.01	0.42	10	0.89	−0.10	−0.15	−0.18*	0.63***	−0.28***	−0.13	−0.18*	−0.32***	
10. T2 Burnout	1–7	1.93	0.71	10	0.82	0.27***	0.36***	0.31***	−0.27***	0.61***	0.32***	0.50***	0.38***	−0.28***

*N* = 166; *p < 0.05; **p < 0.01; ***p < 0.001.

**Figure 1:**
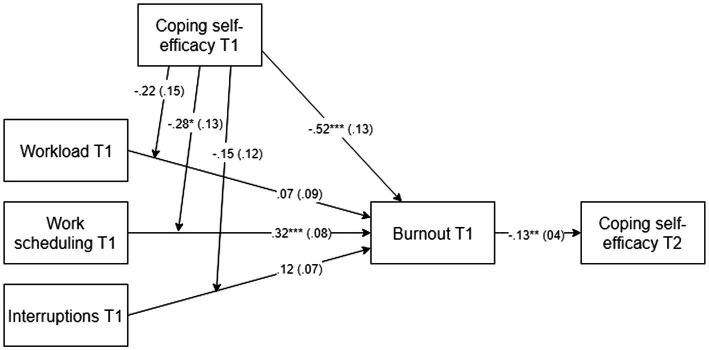
Final model. Direct path coefficients (unstandardised *b*) and moderations are presented with SE in parentheses. *N* = 166, *p < 0.05, **p < 0.01, ***p < 0.001, T1 = Time 1, T2 = Time 2. Results from three separate analyses (per stressor and its interaction with coping self-efficacy) were combined into this model.

The direct paths and moderation results (separate analysis per stressor and its interaction with CSE) are combined in Figure 1. Hypothesis 1, which stated that chronic stressors positively relate to burnout, was partially supported. The results showed that the overall model predicting burnout at T1 was significant (*R*^2^ = 0.36, *F*(5, 160) = 18.29, *p* < 0.001). Burnout at T1 is significantly predicted by work scheduling T1 (*b* = 0.32, *t*(160) = 4.09, *p* < 0.001; H1b). Workload (H1a) and interruptions at T1 (H1c) did not predict burnout at T1 (Figure 1; *b* = 0.07, *t*(160) = 0.75, *p* = 0.456; *b* = 0.12, *t*(160) = 1.74, *p* = 0.084). The same model was re-tested with burnout at T2 as mediator to examine the long-term effects of stressors on burnout. The results were similar; the overall model predicting burnout at T2 was significant (*R*^2^ = 0.18, *F*(5, 160) = 7.26, *p* < 0.001). Burnout at T2 was significantly predicted by work scheduling T1 (*b* = 0.19, *t*(156) = 2.55, *p* = 0.012; H1b). Workload (H1a) and interruptions (H1c) T1 do not predict burnout at T2 (*b* = 0.07, *t*(160) = 0.80, *p* = 0.426; *b* = 0.08, *t*(160) = 1.22, *p* = 0.226).

**Figure 2:**
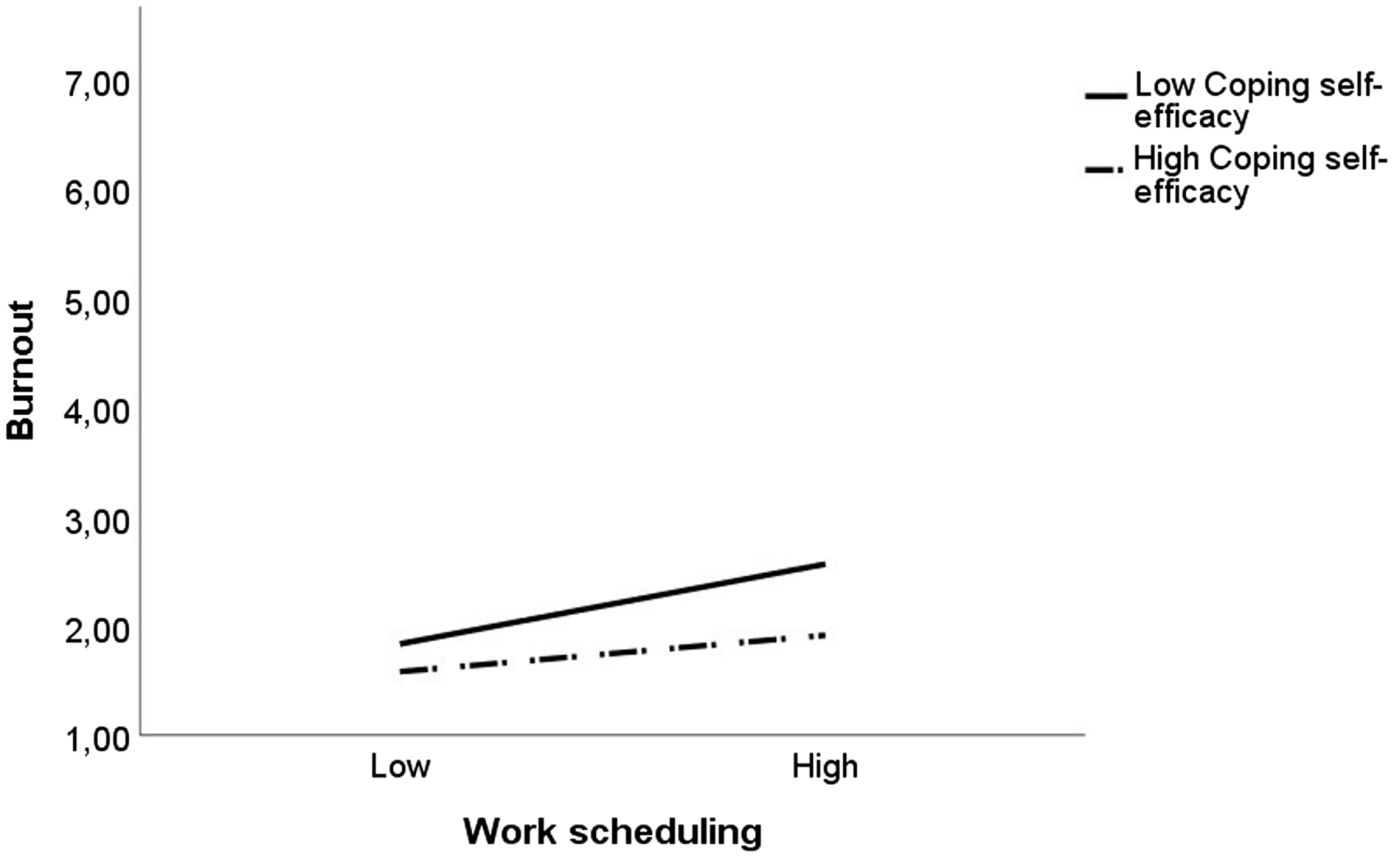
Moderating effect of coping self-efficacy on the relationship between work scheduling and burnout. All predictors in this figure are centred and standardised and measured at T1. Predictor levels: Low = 1 SD below the mean, High = 1 SD above the mean.

Hypothesis 2, which stated that CSE moderates the positive relationship between stressors and burnout, was partially supported. The results showed that for burnout at T1, the interaction between work scheduling and CSE was significant (Δ*R*^2^ = 0.02, *b* = −0.28, *p* = 0.029). *Post hoc* tests of the simple slopes were conducted for the significant interaction (Figure 2). The effect of work scheduling on burnout at low (−1SD; *b* = 0.44, *p* < 0.001) and high levels (+1SD; *b* = 0.20, *p* = 0.038) of CSE indicated that the harmful effect of this stressor was weakened amongst POs with high levels of CSE. No significant interaction effect was found for CSE with workload or with interruptions.

Hypothesis 3, which stated that burnout negatively relates to CSE, was supported. The overall model that predicted CSE at T2 is significant (*R*^2^ = 0.08, *F*(4, 161) = 3.68, *p* = 0.007). CSE at T2 was significantly predicted by burnout T1 (Figure 1; *b* = −0.13, *t*(161) = −2.89, *p* = 0.004). Conclusively, in support of Hypothesis 3, we found that burnout negatively affected CSE over time. Furthermore, there was a significant direct effect of CSE on burnout. CSE at T1 significantly predicted burnout T1 (Figure 1; *b* = −0.52, *t*(160) = −4.01, *p* < 0.001). The repeated analysis with burnout at T2 as a mediator showed similar results. CSE at T1 negatively predicted burnout T2 (*b* = −0.26, *t*(160) = −2.09, *p* = 0.038), implying a long-term negative effect of CSE on burnout.

### Conclusion

Hypotheses 1 and 2 were partially supported by the results of the moderated mediation analyses and Hypothesis 3 was fully supported. We conclude that work scheduling has an adverse effect on POs’ burnout complaints (i.e., high work scheduling increases burnout symptoms), but that this effect can be buffered by CSE. Moreover, burnout has long-term negative effects on CSE and CSE has negative effects on burnout, initiating a potential resource loss cycle.

A possible limitation of this study is that only two waves of data were investigated, which may undermine the value of our conclusions for long-term processes. Moreover, the model was not fully supported in that workload and interruptions did not significantly relate to burnout. Study 2 was performed to validate the results and our research model. Similar analyses were performed in an independent sample of participants who completed three consecutive waves. Thereby, we adhered to the chronic nature of burnout and depletion processes.

## Study 2

### Materials and methods

#### Participants and procedure

For Study 2, a three-wave online questionnaire was administered at the police district in a large city in Netherlands. Data collection took place in May 2019 (T1), March 2020 (T2), and March 2021 (T3). The times and procedures were identical to Study 1.

Only participants who completed three consecutive waves were included in this study, resulting in a sample of 95 participants. The majority of the POs in the sample were over 30 years old (49% between 30 and 45; 45% older than 45 years). Most had received vocational education (44%, 29% higher education; 27% high school). Gender was not registered to reduce traceability to individuals within their teams.

#### Measures

The measures at T1, T2, and T3 were identical to the measures in Study 1. The results of the reliability analyses of all measures are presented in Table 2.

**Table 2:** Descriptive statistics Study 2 (scale, mean, standard deviation, Cronbach’s alpha, and Pearson correlations).

	Range	Mean	SD	*n* items	α	1	2	3	4	5	6	7	8	9	10	11	12	13	14
1. T1 Workload	1–5	2.74	0.59	3	0.68														
2. T1 Work scheduling	1–5	2.53	0.68	5	0.66	0.28**													
3. T1 Interruptions	1–5	2.75	0.93	2	0.75	0.43***	0.55***												
4. T1 Coping self-efficacy	1–5	3.93	0.42	10	0.85	−0.01	−0.12	−0.06											
5. T1 Burnout symptoms	1–7	2.13	0.89	10	0.87	0.28**	0.51***	0.41***	−0.25*										
6. T2 Workload	1–5	2.70	0.66	3	0.74	0.60***	0.10	0.28**	0.00	0.20									
7. T2 Work scheduling	1–5	2.71	0.72	5	0.67	0.12	0.56***	0.27**	−0.09	0.48***	0.10								
8. T2 Interruptions	1–5	3.08	1.00	2	0.76	0.32**	0.30**	0.60***	−0.11	0.37***	0.32**	0.41***							
9. T2 Coping self-efficacy	1–5	3.93	0.47	10	0.89	−0.04	−0.22*	−0.18	0.69***	−0.38***	−0.12	−0.36***	−0.32**						
10. T2 Burnout symptoms	1–7	2.12	0.91	10	0.89	0.13	0.38***	0.31**	−0.26*	0.76***	0.08	0.48***	0.41***	−0.53***					
11. T3 Workload	1–5	2.71	0.58	3	0.58	0.64***	0.26*	0.35***	−0.07	0.17	0.63***	0.07	0.26**	−0.16	0.04				
12. T3 Work scheduling	1–5	2.67	0.73	5	0.71	0.07	0.51***	0.16	−0.15	0.46***	−0.04	0.64***	0.25*	−0.32**	0.40***	0.17			
13. T3 Interruptions	1–5	2.92	0.94	2	0.62	0.30**	0.43***	0.55***	−0.22*	0.37***	0.11	0.37***	0.64***	−0.29**	0.27**	0.15	0.37***		
14. T3 Coping self-efficacy	1–5	3.97	0.46	10	0.89	−0.03	−0.19	−0.14	0.67***	−0.25*	−0.01	−0.20	−0.18	0.72***	−0.28**	−0.16	−0.33**	−0.27**	
15. T3 Burnout symptoms	1–7	2.04	0.96	10	0.92	0.20	0.41***	0.37***	−0.20*	0.66***	0.08	0.52***	0.38***	−0.51***	0.77***	0.20	0.56***	0.36***	−0.41***

*N* = 95; *p < 0.05; **p < 0.01; ***p < 0.001.

#### Statistical analysis

The hypotheses were tested with a bootstrapping analysis (Preacher et al., 2007) using Hayes’ Process 3.4.1 macro (model 7, moderated mediation) with 5.000 resamples (Hayes, 2018). Model 7 is a moderated mediation model in which the moderator only moderates the relationship between the independent variable and the mediator. CSE T3 was the dependent variable, stressors T1 were the main predictors, CSE T1 was the moderator, and burnout T2 was the mediating variable (given H3 – the long-term depletion regarding CSE). Separate analyses were conducted for each predictor, whilst the other stressors were included as covariates, to ensure all interaction variables were analysed. All analyses were performed in SPSS 25.

### Results

The means, standard deviations, Cronbach’s alpha coefficients, and bivariate correlations of the scales at all three timepoints are presented in Table 2. Internal consistencies for the stressors varied from weak to moderate over the three timepoints (0.58–0.76). Strong internal consistencies were found for CSE and burnout (0.85–0.92). Correlations between the chronic stressors workload, work scheduling, and interruptions were low to moderate (Haslam and McGarty, 2003). Burnout showed significant positive correlations with the stressors (excl. workload) and significant negative correlations with CSE at all timepoints.

**Figure 3:**
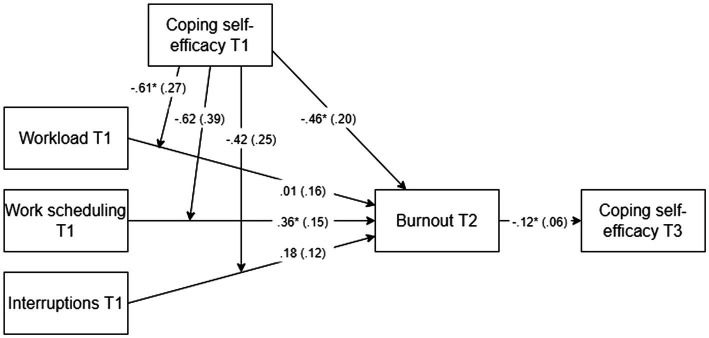
Final model. Direct path coefficients (unstandardised b) and moderations are presented with SE in parentheses. *N* = 95, *p < 0.05, T1 = Time 1, T2 = Time 2, and T3 = Time 3. Results from three separate analyses (per stressor and its interaction with coping self-efficacy) were combined into this model.

The direct paths and moderation results (separate analysis per stressor and its interaction with CSE) are combined in Figure 3. Hypothesis 1,which stated that chronic stressors positively relate to burnout, was partially supported. The results showed that the overall model that predicted burnout at T2 was significant (*R*^2^ = 0.25, *F*(5, 89) = 5.93, *p* < 0.001). Burnout at T2 was significantly predicted by work scheduling at T1 (*b* = 0.36, *t*(89) = 2.37, *p* = 0.020; H1b). Workload (H1a) and interruptions at T1 (H1c) did not predict burnout at T2 (Figure 3; *b* = 0.01, *t*(89) = 0.08, *p* = 0.937; *b* = 0.18, *t*(89) = 1.56, *p* = 0.123).

**Figure 4:**
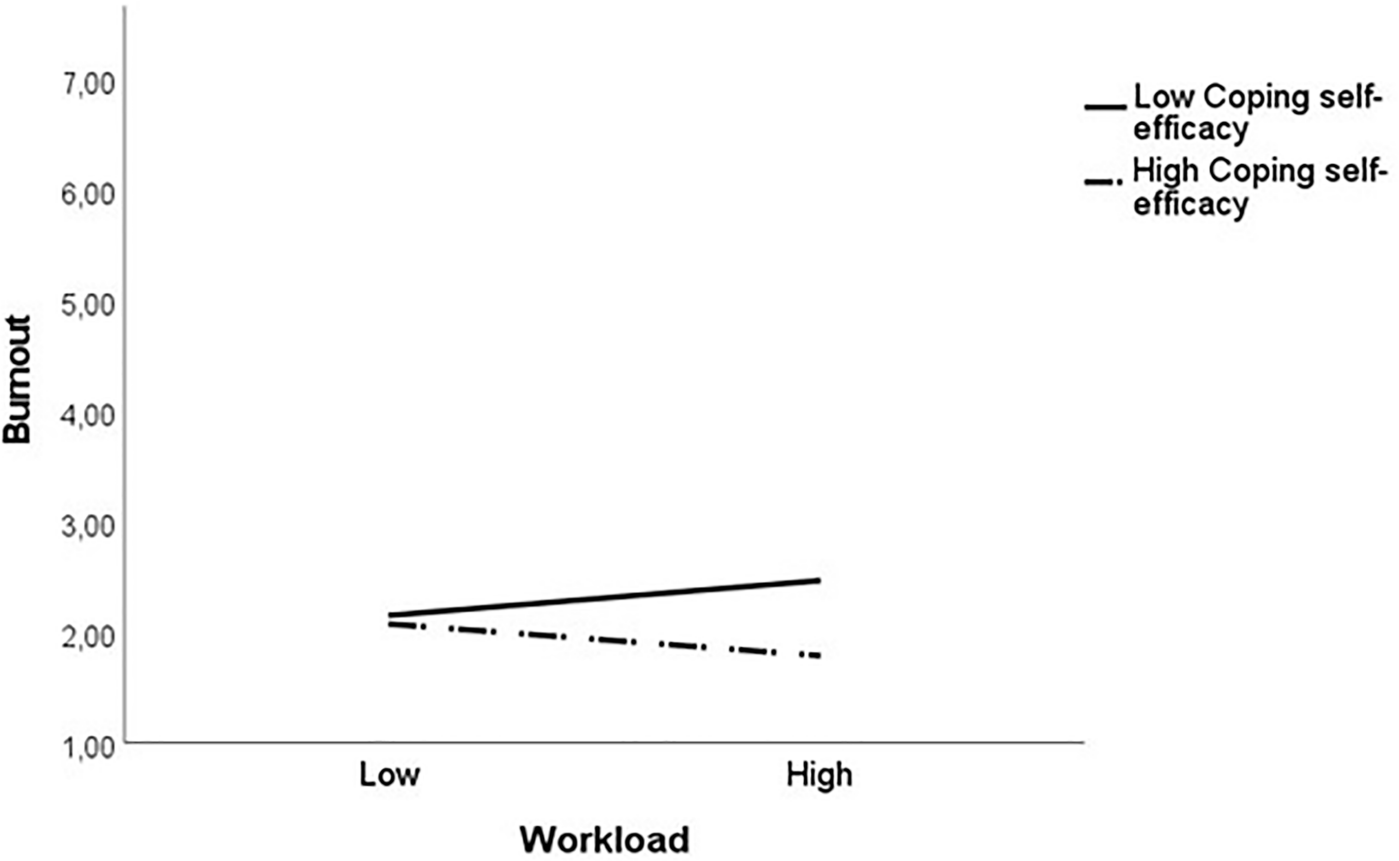
Moderating effect of coping self-efficacy T1 on the relationship between workload T1 and burnout T2. All predictor variables in this figure are centred and standardised. Predictor levels: Low = 1 SD below the mean, High = 1 SD above the mean.

Hypothesis 2, which stated that CSE moderates the positive relationship between stressors and burnout, was partially supported. Results showed that for burnout at T2, the interaction between workload and CSE was significant (Δ*R^2^* = 0.04, *b* = −0.61, *p* = 0.028). *Post hoc* tests of the simple slopes were conducted for the significant interaction (Figure 4). The effect of workload on burnout at low (−1SD; *b* = 0.27, *p* = 0.195) and high levels (+1SD; *b* = −0.24, *p* = 0.195) of CSE indicated that the harmful effect of this stressor was weakened amongst POs with high levels of CSE. No significant interaction effect was found for CSE with work scheduling or with interruptions.

Hypothesis 3, which stated that burnout negatively relates to CSE, was supported. Although the overall model that predicted CSE at T3 is non-significant (*R*^2^ = 0.09, *F*(4, 90) = 2.24, *p* = 0.071), CSE at T3 is significantly predicted by burnout at T2 (Figure 3; *b* = −0.12, *t*(90) = −2.22, *p* = 0.029). Conclusively, in support of Hypothesis 3, we found that burnout negatively affected CSE over time. The non-significance of the overall model seemed to contradict our conclusion. However, Hypothesis 3 only concerned the direct effect of burnout on CSE and is thus supported. Furthermore, there was a significant direct effect of CSE on burnout. CSE at T1 significantly predicted burnout at T2 (Figure 3; *b* = −0.46, *t*(89) = −2.25, *p* = 0.027), implying a negative effect of CSE on burnout.

### Conclusion

The results of Study 2 resemble the results of Study 1. Hypotheses 1 and 2 were partially supported and Hypothesis 3 was fully supported. With Study 2 we confirmed our results from Study 1. That is, CSE buffers the effects of a chronic stressor (i.e., work scheduling, Study 1; workload, Study 2) on burnout and burnout has long-term negative effects on CSE, confirming the depletion hypothesis. However, in Study 2 as well, the model was not fully supported in that workload and interruptions did not significantly relate to burnout. Furthermore, the overall model predicting CSE was not significant.

## Discussion

The aim of this study is to investigate the complex role of CSE as personal coping resource in the chronic stress process. Based on COR theory (Hobfoll, 1989), two studies, one with a two-wave and the second with a three-wave questionnaire design, were used to investigate the role of CSE as a moderator in the long-term stressor-strain relationship (H2) and as an outcome of burnout (H3). In support of the moderation hypothesis, the findings from Study 1 indicated that work scheduling increases burnout symptoms and that CSE weakens this effect. In Study 2, CSE weakened the effect of another chronic stressor (workload) on burnout. Furthermore, in support of the depletion hypothesis, the results showed that burnout has a direct negative effect on CSE at later timepoints, and CSE has a direct negative effect on burnout symptoms. Together, this implies that CSE is important in reducing the risk of burnout, and that burnout is a risk factor for future coping.

The findings on direct effects of chronic stressors on burnout are partially consistent with the literature (Ma et al., 2015; Peterson et al., 2019). Participants who experienced high work scheduling demands also experienced more burnout symptoms. Although other studies have found positive associations between burnout and workload (Demerouti et al., 2004; Van den Broeck et al., 2010; Taris et al., 2017) and interruptions (Lin et al., 2013; Beck et al., 2017), our findings do not support this claim. The moderate to strong correlations amongst the stressors in our studies and possibly the shifted interaction suggest that workload and interruptions contribute to the overall level of organisational demands. It seems that the effects of workload and interruptions on burnout are statistically suppressed when work scheduling is included as a predictor in the same model.

Furthermore, the findings support earlier research on efficacy beliefs and coping resources (Delahaij and Van Dam, 2017). POs who have higher CSE experienced less burnout complaints when work scheduling was high. This provides support for the COR theory (Hobfoll et al., 2018), which states that resources can buffer the health impairment process, especially when demands are high.

Moreover, in line with the depletion hypothesis with CSE as outcome, CSE was lower for POs who experienced higher burnout complaints at earlier timepoints. Altogether, the results imply that if CSE is reduced as a result of increased burnout symptoms, it cannot be invested in buffering the effects of stressors on burnout in the future, which initiates a depletion process of this resource. Results are similar to the findings of a recent study (Otto et al., 2021) which found that personal resources (i.e., self-efficacy and optimism) have a negative reciprocal relationship with burnout. That is, personal resources negatively predicted burnout and burnout negatively predicted personal resources. Our results also corroborate Llorens-Gumbau and Salanova-Soria’s (2014) findings of a loss cycle of self-efficacy through negative affective states. Our findings showed that when CSE is low, individuals are at increased risk of burnout and subsequently at risk of further CSE depletion. Fostering CSE could thus prevent future loss of resources and possibly the onset of a loss cycle, which conforms to COR theory where resources are actively pursued to maintain and increase wellbeing (Hobfoll, 1989; Llorens et al., 2007; Hobfoll et al., 2018).

### Limitations

Some limitations of these studies should be addressed. First, the samples in both studies were relatively small, which may have limited statistical power. Only participants who completed two (Study 1) or three (Study 2) consecutive waves were included for analyses. The generalizability of the effects was also limited due to the sample size. Moreover, the research sample consisted mainly of (psychologically) healthy adults who showed limited signs of burnout. Reproduction of the studies in other police districts, and also amongst burned out populations, could validate the results and increase their generalizability.

Second, although the scales were validated, the studies made use of self-report measures. The aggregated (anonymized) data was reported to team leaders to stimulate communication amongst team members. Although confidentiality was emphasised, participants’ fear of assessment could have resulted in socially desirable answers. However, this bias is perhaps limited because participants were informed about the possible future improvements in the organisation based on the aggregated results.

Third, the use of a two-wave sample for longitudinal mediation analyses can be considered controversial because the relation of the independent variable to the mediator or the relation of the mediator to the dependent variable is cross-sectional (Mac Kinnon et al., 2013). Although the analyses were performed separately with both burnout at T1 and burnout at T2 as mediating variables, the analysis would still include a cross-sectional relation in Study 1. This limitation is reduced when a third wave of data is included, which was the case for Study 2.

### Implications and future research

Research on demands and wellbeing in organisations often refer to resources as a mediator, moderator, or predictor in the stress process (Westman et al., 2004). Recently, research has focused on personal and job resources as initiators of a motivational process that also can act as buffers in the health impairment process, based on the JD-R model (e.g., Demerouti and Bakker, 2011). Contrastingly, research on resources as the outcome or as a recursive aspect of the stress process is scarce. The COR theory provides a theoretical framework for this research where investing or protecting resources can lead to gain cycles or loss cycles. The COR theory can guide interventions to decrease burnout and future research that examines the depletion of coping resources. This study focuses on the depletion of the personal resource CSE because it plays a key role in protecting wellbeing at work. The results indicate that CSE is important in preventing burnout symptoms as a result of exposure to chronic stress and that burnout can negatively affect CSE. Failure to effectively cope with stressors can undermine an individual’s self-efficacy, because burnout-related symptoms (and ineffective coping in itself) can be perceived as an indication of an inability to cope effectively. As such, individuals would benefit from monitoring and gaining coping resources when they are at risk of burnout. In particular, police organisations and POs are recommended to regularly monitor levels of CSE and burnout. Training POs to increase or maintain their CSE (for instance through mastery experiences in simulations) and increasing awareness of burnout symptoms at all levels in police organisations could help prevent burnout and depletion of CSE for POs and further societal impact of police absenteeism and turnover.

Future studies could focus on the individual level or compare healthy and burned out individuals to provide insight into the development of burnout and the role of CSE. The vital questions are: Why do some, but not all individuals, develop psychological complaints? How can we prevent individuals from becoming burned out? How can we train individuals to more automatically use their coping resources to prevent psychological complaints? How can we successfully interrupt and maybe overturn the depletion process after its onset? Also, the tenets of the COR theory allow for personal resources to be examined in different contexts. As opposed to a depletion process, it would be interesting to investigate whether a gain process of CSE (Llorens et al., 2007) exists for individuals (e.g., police officers) and how such a gain process can be initiated.

## Conclusion

In summary, the results from these studies imply that a depletion process of CSE exists as a result of burnout symptoms. Dutch police organisations should monitor and aim to reduce work stressors, and in particular, work scheduling. Also, they should provide POs opportunities to maintain and train their CSE to reduce the risk of experiencing burnout-related complaints as well as the risk of depleting their CSE in the long-term.

## Data availability statement

The datasets presented in this article are not readily available because the nature of this research, the participating organisation and individual participants of this study did not agree for their data to be shared publicly. Requests to access the datasets should be directed to LE, liselotte.eikenhout@tno.nl.

## Ethics statement

The studies involving human participants were reviewed and approved by TNO internal review board registration number: 2018-075. The patients/participants provided their written informed consent to participate in this study.

## Author contributions

LE is the main researcher and author of the paper. RD, KD, WK, IH, and JR have a supervisory and review role. All authors contributed to the article and approved the submitted version.

## Funding

Funding for open access publication of this article was provided by the Library Committee Open Universiteit – OA Fund.

## Conflict of interest

The authors declare that the research was conducted in the absence of any commercial or financial relationships that could be construed as a potential conflict of interest.

## Publisher’s note

All claims expressed in this article are solely those of the authors and do not necessarily represent those of their affiliated organizations, or those of the publisher, the editors and the reviewers. Any product that may be evaluated in this article, or claim that may be made by its manufacturer, is not guaranteed or endorsed by the publisher.

## Footnotes

^1^Kamphuis et al. (2014) is a Dutch internal report on the Police Resilience Monitor. The report and the items used in this study (in Dutch) are available upon request to the first author.

## References

[ref1] Abdollahi M. K. (2002). Understanding police stress research. J. Forensic Psychol. Pract. 2, 1–24. doi: 10.1300/J158v02n02_01

[ref2] American Psychological Association (2017). Ethical Principles of Psychologists and Code of Conduct American Psychological Association Available at: https://www.apa.org/ethics/code

[ref3] Anshel M. H. (2000). A conceptual model and implications for coping with stressful events in police work. Crim. Justice Behav. 27, 375–400. doi: 10.1177/0093854800027003006

[ref4] Bakker A. B. Heuven E. (2006). Emotional dissonance, burnout, and in-role performance among nurses and police officers. Int. J. Stress. Manag. 13, 423–440. doi: 10.1037/1072-5245.13.4.423

[ref5] Bandura A. (1997). Self-efficacy: The Exercise of Control. New York, US: W H Freeman and Company.

[ref6] Bandura A. (2001). Social cognitive theory: an agentic perspective. Annu. Rev. Psychol. 52, 1–26. doi: 10.1146/annurev.psych.52.1.1, PMID: 11148297

[ref7] Beck J. W. Scholer A. A. Hughes J. (2017). Divergent effects of distance versus velocity disturbances on emotional experiences during goal pursuit. J. Appl. Psychol. 102, 1109–1123. doi: 10.1037/apl0000210, PMID: 28333496

[ref8] Benight C. C. Bandura A. (2004). Social cognitive theory of posttraumatic recovery: the role of perceived self-efficacy. Behav. Res. Ther. 42, 1129–1148. doi: 10.1016/j.brat.2003.08.008, PMID: 15350854

[ref9] Benight C. C. Harper M. L. (2002). Coping self-efficacy perceptions as a mediator between acute stress response and long-term distress following natural disasters. J. Trauma. Stress. 15, 177–186. doi: 10.1023/A:1015295025950, PMID: 12092909

[ref10] Burns R. A. Butterworth P. Anstey K. J. (2016). An examination of the long-term impact of job strain on mental health and wellbeing over a 12-year period. Soc. Psychiatry Psychiatr. Epidemiol. 51, 725–733. doi: 10.1007/s00127-016-1192-9, PMID: 26875152

[ref11] Day A. L. Livingstone H. A. (2001). Chronic and acute stressors among military personnel: do coping styles buffer their negative impact on health? J. Occup. Health Psychol. 6, 348–360. doi: 10.1037/1076-8998.6.4.348, PMID: 11605828

[ref12] Delahaij R. Van Dam K. (2017). Coping with acute stress in the military: the influence of coping style, coping self-efficacy and appraisal emotions. Personal. Individ. Differ. 119, 13–18. doi: 10.1016/j.paid.2017.06.021

[ref13] Delahaij R. Van Dam K. Gaillard A. W. K. Soeters J. (2011). Predicting performance under acute stress: the role of individual characteristics. Int. J. Stress. Manag. 18, 49–66. doi: 10.1037/a0020891

[ref14] Demerouti E. Bakker A. B. (2011). The job demands–resources model: challenges for future research. SA J. Ind. Psychol. 37:9. doi: 10.4102/sajip.v37i2.974

[ref15] Demerouti E. Bakker A. B. Bulters A. J. (2004). The loss spiral of work pressure, work–home interference and exhaustion: reciprocal relations in a three-wave study. J. Vocat. Behav. 64, 131–149. doi: 10.1016/S0001-8791(03)00030-7

[ref16] Eckenrode J. (1984). Impact of chronic and acute stressors on daily reports of mood. J. Pers. Soc. Psychol. 46, 907–918. doi: 10.1037/0022-3514.46.4.907, PMID: 6737199

[ref17] Folkman S. Lazarus R. S. (1985). If it changes it must be a process: study of emotion and coping during three stages of a college examination. J. Pers. Soc. Psychol. 48, 150–170. doi: 10.1037/0022-3514.48.1.150, PMID: 2980281

[ref18] Folkman S. Lazarus R. S. Gruen R. J. DeLongis A. (1986). Appraisal, coping, health status, and psychological symptoms. J. Pers. Soc. Psychol. 50, 571–579. doi: 10.1037/0022-3514.50.3.571 3701593

[ref19] Gerber M. Hartmann T. Brand S. Holsboer-Trachsler E. Pühse U. (2010). The relationship between shift work, perceived stress, sleep and health in Swiss police officers. J. Crim. Just. 38, 1167–1175. doi: 10.1016/j.jcrimjus.2010.09.005

[ref20] Goldstein D. S. Kopin I. J. (2007). Evolution of concepts of stress. Stress 10, 109–120. doi: 10.1080/10253890701288935 17514579

[ref21] Haslam S. A. McGarty C. (2003). Research Methods and Statistics in Psychology. London, UK: Sage Publications.

[ref22] Hayes A. F. (2018). Introduction to Mediation, Moderation, and Conditional Process Analysis, second edition: A Regression-Based Approach Guilford Press.

[ref23] Hobfoll S. E. (1989). Conservation of resources: a new attempt at conceptualizing stress. Am. Psychol. 44, 513–524. doi: 10.1037/0003-066X.44.3.513, PMID: 2648906

[ref24] Hobfoll S. E. Halbesleben J. Neveu J.-P. Westman M. (2018). Conservation of resources in the organizational context: the reality of resources and their consequences. Annu. Rev. Organ. Psych. Organ. Behav. 5, 103–128. doi: 10.1146/annurev-orgpsych-032117-104640

[ref25] Hooftman W. E. Mars G. M. J. Janssen B. De Vroome E. M. M. Ramaekers M. M. M. J. Van den Bossche S. N. J. (2018). Nationale Enquête Arbeidsomstandigheden 2017. Methodologie en globale resultaten. TNO/CBS. Available at: http://resolver.tudelft.nl/uuid:43ee0e24-f538-429b-9a85-5a63fc6b0875

[ref26] Huijs J. J. J. M. Houtman I. L. D. Kallen V. L. (2014). Langdurig verzuim bij de Nederlandse politie (TNO 2014 R14079). TNO. Available at: http://resolver.tudelft.nl/uuid:a1f83bfd-31cc-4d6d-b27f-e0af2d15aa57

[ref27] Igic I. Keller A. C. Elfering A. Tschan F. Kälin W. Semmer N. K. (2017). Ten-year trajectories of stressors and resources at work: cumulative and chronic effects on health and well-being. J. Appl. Psychol. 102, 1317–1343. doi: 10.1037/apl0000225, PMID: 28447833

[ref28] Kamphuis W. Delahaij R. Preenen P. (2014). Validatie weerbaarheidsmonitor Politie–basis Politie Zorg [validation of the Resilience Monitor for the Police–Basic Police Teams] (TNO 2014 M11035). Soesterberg, NL: TNO.

[ref29] Keller A. C. Meier L. L. Elfering A. Semmer N. K. (2020). Please wait until I am done! Longitudinal effects of work interruptions on employee well-being. Work Stress 34, 148–167. doi: 10.1080/02678373.2019.1579266

[ref30] Kop N. Euwema M. C. (2001). Occupational stress and the use of force by Dutch police officers. Crim. Justice Behav. 28, 631–652. doi: 10.1177/009385480102800505

[ref31] Lan T. Chen M. Zeng X. Liu T. (2020). The influence of job and individual resources on work engagement among Chinese police officers: a moderated mediation model. Front. Psychol. 11:497. doi: 10.3389/fpsyg.2020.00497, PMID: 32317999 PMC7154180

[ref32] Lazarus R. S. (1991). Emotion and Adaptation. New York, US: Oxford University Press.

[ref33] Lee R. T. Ashforth B. E. (1996). A meta-analytic examination of the correlates of the three dimensions of job burnout. J. Appl. Psychol. 81, 123–133. doi: 10.1037/0021-9010.81.2.123, PMID: 8603909

[ref34] Lepore S. J. Miles H. J. Levy J. S. (1997). Relation of chronic and episodic stressors to psychological distress, reactivity, and health problems. Int. J. Behav. Med. 4, 39–59. doi: 10.1207/s15327558ijbm0401_3, PMID: 16250741

[ref35] Lin B. C. Kain J. M. Fritz C. (2013). Don’t interrupt me! An examination of the relationship between intrusions at work and employee strain. Int. J. Stress. Manag. 20, 77–94. doi: 10.1037/a0031637

[ref36] Llorens S. Schaufeli W. Bakker A. Salanova M. (2007). Does a positive gain spiral of resources, efficacy beliefs and engagement exist? Comput. Hum. Behav. 23, 825–841. doi: 10.1016/j.chb.2004.11.012

[ref37] Llorens-Gumbau S. Salanova-Soria M. (2014). Loss and gain cycles? A longitudinal study about burnout, engagement and self-efficacy. Burn. Res. 1, 3–11. doi: 10.1016/j.burn.2014.02.001

[ref38] Ma C. C. Andrew M. E. Fekedulegn D. Gu J. K. Hartley T. A. Charles L. E.. (2015). Shift work and occupational stress in police officers. Saf. Health Work 6, 25–29. doi: 10.1016/j.shaw.2014.10.001, PMID: 25830066 PMC4372186

[ref39] Mac Kinnon D. P. Kisbu-Sakarya Y. Gottschall A. C. (2013). “Developments in mediation analysis” in The Oxford Handbook of Quantitative Methods: Statistical Analysis, vol. 2. ed.T. D. Little (New York, US: Oxford University Press)

[ref40] Meijman T. F. Mulder G. (1998). “Psychological aspects of workload” in A Handbook of Work and Organizational Psychology: Vol. 2 Work Psychology. eds. Thierry H. Wolff C. D. Drenth P. J. D. (East Sussex, UK: Psychology Press Ltd), 5–33.

[ref41] NOS. (2019). Klokkenluider hekelt ‘lelijk gedrag’ politietop, korpschef geschrokken. Available at: https://nos.nl/l/2293256

[ref42] Otto M. C. B. Van Ruysseveldt J. Hoefsmit N. Van Dam K. (2021). Examining the mediating role of resources in the temporal relationship between proactive burnout prevention and burnout. BMC Public Health 21:599. doi: 10.1186/s12889-021-10670-7, PMID: 33771155 PMC8004439

[ref43] Peterson S. A. Wolkow A. P. Lockley S. W. O’Brien C. S. Qadri S. Sullivan J. P.. (2019). Associations between shift work characteristics, shift work schedules, sleep and burnout in north American police officers: a cross-sectional study. BMJ Open 9:e030302. doi: 10.1136/bmjopen-2019-030302, PMID: 31791964 PMC6924705

[ref44] Politie N. L. (2021). Jaarverantwoording politie 2020 [annual report police 2020] [annual report]. Available at: https://www.politie.nl/publicaties

[ref45] Preacher K. J. Rucker D. D. Hayes A. F. (2007). Addressing moderated mediation hypotheses: theory, methods, and prescriptions. Multivar. Behav. Res. 42, 185–227. doi: 10.1080/00273170701341316, PMID: 26821081

[ref46] Puranik H. Koopman J. Vough H. C. (2020). Pardon the interruption: an integrative review and future research agenda for research on work interruptions. J. Manag. 46, 806–842. doi: 10.1177/0149206319887428

[ref47] Queirós C. Kaiseler M. DaSilva A. L. (2013). Burnout as a predictor of aggressivity among police officers. Eur. J. Policing Stud. 1, 110–134. doi: 10.5553/EJPS/2034760X2013001002003

[ref48] Queirós C. Passos F. Bártolo A. Marques A. J. da Silva C. F. Pereira A. (2020). Burnout and stress measurement in police officers: literature review and a study with the operational police stress questionnaire. Front. Psychol. 11:587. doi: 10.3389/fpsyg.2020.00587, PMID: 32457673 PMC7221164

[ref49] Schaufeli W. B. Bakker A. B. Van Rhenen W. (2009). How changes in job demands and resources predict burnout, work engagement, and sickness absenteeism. J. Organ. Behav. 30, 893–917. doi: 10.1002/job.595

[ref50] Schaufeli W. B. De Witte H. Desart S. (2019). Handleiding Burnout Assessment Tool (BAT) [Internal Report, Manual]. Leuven, BE: KU Leuven.

[ref51] Schaufeli W. B. Taris T. W. (2014). “A critical review of the job demands-resources model: implications for improving work and health” in Bridging occupational, organizational and public health: A Transdisciplinary approach. eds. Bauer G. F. Hämmig O. (Netherlands: Springer), 43–68.

[ref52] Schaufeli W. B. Van Dierendonck D. (1995). A cautionary note about the cross-national and clinical validity of cut-off points for the Maslach burnout inventory. Psychol. Rep. 76, 1083–1090. doi: 10.2466/pr0.1995.76.3c.1083, PMID: 7480470

[ref53] Smith A. J. Benight C. C. Cieslak R. (2013). Social support and postdeployment coping self-efficacy as predictors of distress among combat veterans. Mil. Psychol. 25, 452–461. doi: 10.1037/mil0000013

[ref54] Stetz T. A. Stetz M. C. Bliese P. D. (2006). The importance of self-efficacy in the moderating effects of social support on stressor–strain relationships. Work Stress 20, 49–59. doi: 10.1080/02678370600624039

[ref55] Taris T. W. Ybema J. F. Beek I. V. (2017). Burnout and engagement: identical twins or just close relatives? Burn. Res. 5, 3–11. doi: 10.1016/j.burn.2017.05.002

[ref56] Van den Broeck A. De Cuyper N. De Witte H. Vansteenkiste M. (2010). Not all job demands are equal: differentiating job hindrances and job challenges in the job demands–resources model. Eur. J. Work Organ. Psy. 19, 735–759. doi: 10.1080/13594320903223839

[ref57] Violanti J. M. Charles L. E. McCanlies E. Hartley T. A. Baughman P. Andrew M. E.. (2017). Police stressors and health: a state-of-the-art review. Policing (Bradford, England) 40, 642–656. doi: 10.1108/PIJPSM-06-2016-0097, PMID: 30846905 PMC6400077

[ref58] Westman M. Hobfoll S. E. Chen S. Davidson O. B. Laski S. (2004). “Organizational stress through the lens of conservation of resources (COR) theory” in Exploring Interpersonal Dynamics. eds. Perrewe P. L. Ganster D. C., vol. 4 (Bingley, UK: Emerald Group Publishing Limited), 167–220.

[ref59] Zeng X. Zhang X. Chen M. Liu J. Wu C. (2020). The influence of perceived organizational support on police job burnout: a moderated mediation model. Front. Psychol. 11:948. doi: 10.3389/fpsyg.2020.00948, PMID: 32528368 PMC7265159

